# Evaluation on curative effects of ethylene diamine tetra-acetic acid chelation therapy in treating with atherosclerotic cardiovascular disease: A protocol for systematic review and meta-analysis

**DOI:** 10.1097/MD.0000000000023346

**Published:** 2020-12-24

**Authors:** Tao Song, Daimin Zhang

**Affiliations:** a Department of Cardiology, People's Hospital of Lanling County, Linyi, Shandong; b Department of Cardiology, Nanjing First Hospital, Nanjing Medical University, Nanjing, Jiangsu, China.

**Keywords:** cardiovascular disease, chelation therapy, ethylene diamine tetra-acetic acid, systematic review

## Abstract

**Background:**

Ethylene diamine tetra-acetic acid (EDTA) is a chelating agent which attach to metals such as calcium and enables their elimination. In particular, some researchers suggest chelation with EDTA to treat cardiovascular disease with the hypothesis of moderating calcium to decrease atherosclerotic calcification of arteries. However, chelation with EDTA therapeutic effects in atherosclerotic cardiovascular disease is still unclear. Therefore, we propose to undertake a meta-analysis to assess the curative effects of EDTA chelation therapy in patients with atherosclerotic cardiovascular disease.

**Methods:**

In the current study, we set to perform a systematic literature search using the electronic databases of 4 most commonly used English databases (EMBASE, MEDLINE, Cochrane Library, and ClinicalTrials.gov trials register), as well as 3 most commonly employed Chinese databases (China Nation Knowledge Infrastructure, Wan Fang, and VIP), from the date of database inception until September 30, 2020 to identify relevant randomized controlled studies of the evaluation on curative effects of EDTA chelation therapy in patients with atherosclerotic cardiovascular disease. In the study, 2 authors worked independently to screen search results, chose studies for inclusion, then they extracted pertinent data to evaluate and study quality based on Cochrane Risk of Bias Tool V.2.0. Additionally, we will address discrepancies by consultation with a third author. We also intend to use pooled risk ratio (RR) and pooled mean difference (MD) or pooled standardized mean difference (SMD) with 95% confidence intervals (CI) to approximate the relative strength of curative effects of EDTA chelation therapy in patients with atherosclerotic cardiovascular disease.

**Results:**

The results of the current study will systematically assess curative effects of EDTA chelation therapy in patients with atherosclerotic cardiovascular disease.

**Conclusion:**

The study will infer the currently published evidence to evaluate curative effects of EDTA chelation therapy in patients with atherosclerotic cardiovascular disease, which might be beneficial to these patients.

**Ethics and dissemination:**

The present study is a systematic review, hence the pooled results are founded upon the published evidence. Therefore, ethical approval is not necessary for the study.

**Open Science Framework Registration Number:**

October 20, 2020.osf.io/tvmk8. (https://osf.io/tvmk8/).

## 1 Introduction

Atherosclerotic cardiovascular disease encompasses build-up of cholesterol inscriptions in the arteries which result in myocardial infarction, ischemic cerebrovascular diseases (ischemic stroke/transient ischemic attack), angina, and peripheral arterial disease.^[1]^ Atherosclerotic cardiovascular disease is considered one of the main causes of incidences and mortalities worldwide, and the number of patients with atherosclerotic cardiovascular disease has continued to increase over the rent years.^[2]^ An estimated 80% of the atherosclerotic cardiovascular disease burden ensues in many countries, particularly in poorer countries.^[2,3]^ Its causes are complex and closely associated with risk factors such as age, genetic factors, unhealthy lifestyle, and subclinical disease markers.^[4,5]^ The interaction of these risk factors may ultimately lead to atherosclerotic cardiovascular. Therefore, it is needful to put in place measures to prevent some of these comprehensive factors.

Chelation therapy to remove heavy metals from the body was originally described in the 1940s in response to lewisite. Subsequently, Clarke et al was the first to describe the role of chelation therapy using ethylene diamine tetra-acetic acid (EDTA) in the treatment of stable angina in 1956. He found remarkably improved outcomes in 19 out of 20 participants.^[6]^ Studies evaluating curative effects of EDTA chelation therapy in patients with atherosclerotic cardiovascular disease are limited. Also, the results are still controversial. In this study, we will collect available published literature of EDTA chelation therapy and atherosclerotic cardiovascular disease for the present study, to evaluate curative effects of EDTA chelation therapy in patients with atherosclerotic cardiovascular disease and establish a new evidence for the control and prevention of atherosclerotic cardiovascular disease.

## 2 Methods

This protocol observes the Preferred Reporting Items for Systematic Review and Meta-analysis Protocols (PRISMA-P) guidelines.^[7]^ Accordingly, the study was registered at the Open Science Framework (OSF) on October 20, 2020. The registration DOI number of the present study is 10.17605/OSF.IO/TVMK8.

## 3 Criteria for considering studies for this review

### 3.1 Types of studies

In the current study, we will incorporate randomized controlled trials (RCTs) of the evaluation on curative effects of EDTA chelation therapy in patients with atherosclerotic cardiovascular disease.

### 3.2 Types of participants

We will include studies in participants with atherosclerotic cardiovascular disease. No limitations were applied to gender, race, economic status, and educational background.

### 3.3 Types of interventions

RCTs comparing EDTA chelation therapy with placebo, no intervention, and other intervention approaches. We will include all of the durations of treatment.

### 3.4 Types of outcome measures

#### 3.4.1 Major outcomes

From the study, the anticipated major outcomes will be coronary heart disease (CHD) deaths, vascular deaths, and all-cause mortality.

#### 3.4.2 Minor outcomes

The expected minor outcomes of the study will include participant symptoms, indirect and direct test of disease severity, length of hospital stay, non-fatal events (such as myocardial infarction and unstable angina pectoris), fatal events, and unfavorable events such as serious adverse events, as well as not-seriously unpleasant events.

## 4 Search methods for identification of studies

### 4.1 Electronic searches

In this study, we will perform systematic literature search using electronic databases, including 4 most commonly used English databases (EMBASE, MEDLINE, Cochrane Library, and ClinicalTrials.gov trials register) and 3 most frequently utilized Chinese databases (China Nation Knowledge Infrastructure, WanFang, and VIP), from the date of database inception until September 30, 2020 to recognize eligible studies, devoid of any language limitations. We will use the following free text and synonyms to search databases: EDTA, “cardiovascular disease”, “ethylene diamine tetra-acetic acid” together with specialized filters for RCTs

### 4.2 Searching other resources

To accomplish additional eligible studies, we will manually examine the list primary references and carry out citation tracking of included studies.

## 5 Data collection and analysis

### 5.1 Selection of studies

**Figure 1:**
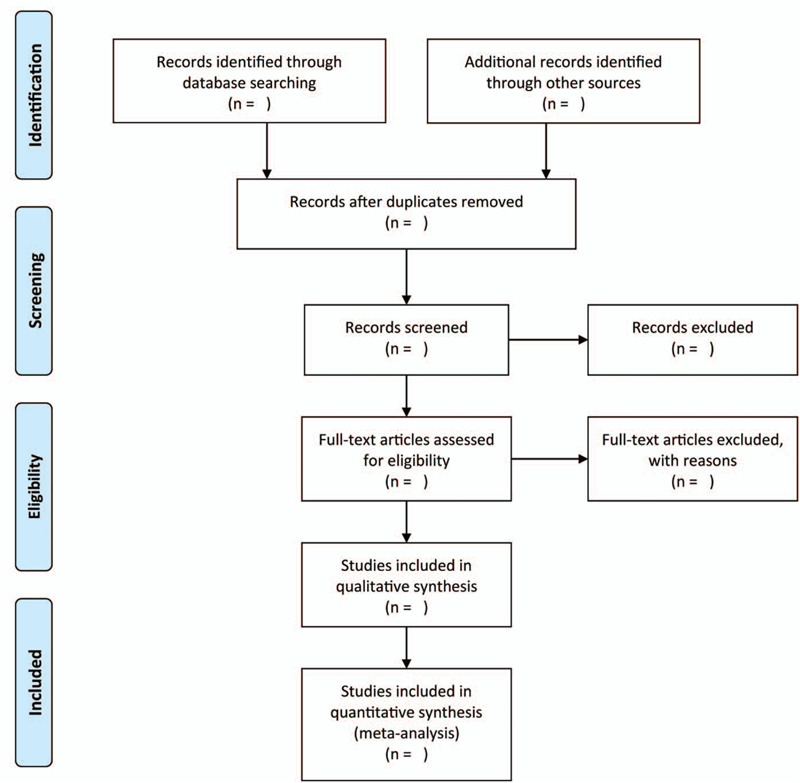
Flow diagram of the literature search.

We will identify studies using Endnote X9 software. Then, 2 independent authors will screen all records from titles and abstracts of all potentially relevant studies via the searches and remove all irrelevant literatures. Any disagreements in opinion between the authors will be resolved by discussion until a consensus is reached. The details of the study selection is illustrated in Figure 1.

### 5.2 Data extraction and management

Two independent authors will review the selected studies and extract data impartially by using data extraction forms specially designed for the extraction of data. We will then extract the following information from the selected studies: publication items, design and methods, participants baseline characteristics, interventions, comparisons, and outcomes of each study. Where disagreements are identified, a third party will be consulted to resolve them until the authors reach a consensus.

### 5.3 Assessment of risk of bias in included studies

The risk of bias will be assessed independently by 2 using the Cochrane Collaborations tool.^[8]^ They will evaluate different sources of bias, including randomization process (selection bias evaluation), allocation concealment process (selection bias evaluation), binding of participants, as well as care personnel (performance bias evaluation), binding of outcome assessors (detection bias evaluation), incomplete outcome data bias, and selective reporting bias, among others. Further, the study will grade each domain as having “low risk”, “high risk”, or “unclear risk” on the basis of the description of incorporated studies. In case of discontentment or disputes between the 2 authors, a third party will be involved to resolve the issue through discussion and to reach a consensus

### 5.4 Measures of treatment effect

We will calculate the mean difference (MD) or standardized mean difference (SMD) with 95% confidence intervals (CI) for the continuous outcomes. We will further use the risk ratio with 95% CI for the dichotomous outcomes.

### 5.5 Dealing with missing data

Missing data will be gathered from the corresponding author where probable. However, if not this is possible, this study will be removed.

### 5.6 Assessment of heterogeneity

The *Chi*^*2*^ statistic and *I*^2^ test will be employed to estimate the heterogeneity of the selected studies. Furthermore, we will employ the random-effect model to pool the data when *I*^2^ is greater than 50% or *P* value is less than .10. Accordingly, we will utilize the fixed-model to pool the data when *I*^2^ is less than 50% or *P* value is greater than .10.

### 5.7 Assessment of reporting biases

Accordingly, we will use Funnel plots and Egger test when at least 10 studies are included in the present study to evaluate the potential reporting bias.^[9,10]^

### 5.8 Assessment of sensitivity analysis

If we identify sufficient studies, we will repeat the analyses while excluding studies with high risks of bias.

## 6 Discussion

Ethylene diamine tetra-acetic acid (EDTA) is a chelating agent binding to calcium, lead, iron, and copper, among other metal ions to form solvable complexes and facilitate their urinary excretion.^[11]^ EDTA chelation therapy has been utilized in patients with atherosclerotic cardiovascular disease on the foundation that calcium chelation could alleviate atherosclerotic plaque with calcium. However, the current pool of evidence of EDTA chelation therapy to treat in atherosclerotic cardiovascular disease remains controversial. To this end, the present study will be carried out when there are enough high-quality literatures and to afford stronger evidence for the prevention of atherosclerotic cardiovascular disease

## Author contributions

**Conceptualization:** Tao Song.

**Data curation:** Tao Song.

**Formal analysis:** Tao Song.

**Investigation:** daiming zhang.

**Methodology:** daiming zhang.

**Writing – original draft:** daiming zhang.

**Writing – review & editing:** daiming zhang.
